# Whole-Transcriptome Analysis of Differentially Expressed Genes in the Vegetative Buds, Floral Buds and Buds of *Chrysanthemum morifolium*

**DOI:** 10.1371/journal.pone.0128009

**Published:** 2015-05-26

**Authors:** Hua Liu, Ming Sun, Dongliang Du, Huitang Pan, Tangren Cheng, Jia Wang, Qixiang Zhang

**Affiliations:** Beijing Key Laboratory of Ornamental Germplasm Innovation and Molecular Breeding, National Engineering Research Center for Floriculture, Beijing Laboratory of Urban and rural ecological environment, College of Landscape Architecture, Beijing Forestry University, Beijing, 100083, China; University of Naples Federico II, ITALY

## Abstract

**Background:**

*Chrysanthemum morifolium* is an important floral crop that is cultivated worldwide. However, due to a lack of genomic resources, very little information is available concerning the molecular mechanisms of flower development in chrysanthemum.

**Results:**

The transcriptomes of chrysanthemum vegetative buds, floral buds and buds were sequenced using Illumina paired-end sequencing technology. A total of 15.4 Gb of reads were assembled into 91,367 unigenes with an average length of 739 bp. A total of 43,137 unigenes showed similarity to known proteins in the Swissprot or NCBI non-redundant protein databases. Additionally, 25,424, 24,321 and 13,704 unigenes were assigned to 56 gene ontology (GO) categories, 25 EuKaryotic Orthologous Groups (KOG) categories, and 285 Kyoto Encyclopedia of Genes and Genomes (KEGG) pathways, respectively. A total of 1,876 differentially expressed genes (DEGs) (1,516 up-regulated, 360 down-regulated) were identified between vegetative buds and floral buds, and 3,300 DEGs (1,277 up-regulated, 1,706 down-regulated) were identified between floral buds and buds. Many genes encoding important transcription factors (e.g., AP2, MYB, MYC, WRKY, NAC and CRT) as well as proteins involved in carbohydrate metabolism, protein kinase activity, plant hormone signal transduction, and the defense responses, among others, were considerably up-regulated in floral buds. Genes involved in the photoperiod pathway and flower organ determination were also identified. These genes represent important candidate genes for molecular cloning and functional analysis to study flowering regulation in chrysanthemum.

**Conclusion:**

This comparative transcriptome analysis revealed significant differences in gene expression and signaling pathway components between the vegetative buds, floral buds and buds of *Chrysanthemum morifolium*. A wide range of genes was implicated in regulating the phase transition from vegetative to reproductive growth. These results should aid researchers in the study of flower-time regulation, breeding and molecular biology in chrysanthemum.

## Introduction

Asteraceae belongs to the euasterids II clade of the core eudicots, one of the largest families of flowering plants. The head-like inflorescence (capitulum) of Asteraceae resembles a single large flower, and this trait is considered to be the key innovation behind the evolutionary success of the Asteraceae [1]. Thus, the trait has been the subject of many phylogenetic and biological studies. In recent years, molecular genetic research on flower development has been restricted to *Gebera hybrida* and *Helianthus annuus* in Asteraceae. *Chrysanthemum morifolium*, another species of Asterceae, is an important crop that is widely cultivated, and indeed, complete year-round production of chrysanthemums is necessary to meet the demands of a growing market [2]. However, the flowering mechanisms and molecular pathways of chrysanthemums involved in flower development have not been well characterized to date due to the lack of genomic information [2].

In temperate regions, the duration of the daily light period, also called the photoperiod, is one of the most important factors controlling flowering time [3]. In Arabidopsis, many genes involved in daylength response have been identified through molecular-genetic approaches, including the genes encoding regulatory proteins for the regulation of flowering, light signal transduction pathways and circadian clock function, such as *CRYPTOCHROME2/FHA* (*CRY2*), *GIGANTEA* (*GI*), *TIMING OF CAB 1* (*TOC1*) and *CONSTANS* (*CO*) [3]. Some important downstream regulatory genes have been identified in the photoperiod pathway. For example, the crucial regulator LEAFY (LFY) functions in floral specification. LFY exhibits weak expression in vegetative tissues, and its expression is significantly up-regulated after receiving photoperiodic signals regulated by the FT pathway and gibberellins [4, 5]. Many MADS-Box transcription factors members, along with APETALA2 (AP2), also play important roles in floral initiation, such as *SUPPRESSOR OF CONSTANS1* (*SOC1*), *SHORT VEGETATIVE PHASE* (*SVP*), *LEAFY* (*LFY*), *APETALA1* (*AP1*), *GIGANTEA* (*GI*), *AGAMOUS* (*AG*) and *PISTILLATA* (*PI*). After the initial expression of *LFY*, *AP1* is expressed throughout the floral meristem, and together with *AP2*, specifies sepal identity [4, 6]. Various feedback mechanisms are used to regulate the actions of these genes in floral meristem specification and in the transition to flowering that occurs by promoting floral initiation and repressing vegetative growth [4]. The classic ABC model includes A-class genes (*AP1*, *AP2*), which determine sepal identity, A-class genes together with B-class genes (*AP3*, *PI*), which determine petals, B-class genes together with a C-class gene (*AG*), which specify stamens, and the C-class gene, which specifies carpel identity [7].

Chrysanthemum is a typical short-day plant. Under short-day treatment, chrysanthemum undergoes a major physiological change and morphological programs as it transits from a vegetative growth period to a reproductive period and finally blooms. This process is regulated by a combination of endogenous and environmental signals. By morphological observation, three developmental periods can be identified during the developmental process from vegetative growth to reproductive growth, including vegetative bud growth, flower bud differentiation, and bud development. In chrysanthemum, only a handful of important transcription factors involved in the flower developmental pathway have been isolated and analyzed, including the homologous genes *APETALA1*, *SEPALLATA3*, and *FRUITFULL* [8]. Therefore, the identification of novel genes and molecular regulatory pathways with important functions will be necessary to improve year-round production techniques in chrysanthemum.

Transcription expression profiling, data assembly, and analysis can provide broad and deep insights into gene regulatory networks and biological pathways because they can reveal the genes downstream of key transcription factors in related pathways [9]. The use of RNA-Seq in the studies of chrysanthemum and related species has been demonstrated [10–12]. However, to date, no transcriptome information concerning the different flower developmental periods of chrysanthemum has been reported. In this study, transcriptional sequencing and analysis of vegetative buds (the apical buds during vegetative growth), floral buds (the apical buds during flower differentiation) and buds of chrysanthemum were performed using Illumina assembly technology and RNA-Seq quantification analysis. Based on transcriptional sequencing and analysis, we first identified differentially expressed genes (DEGs) between the vegetative buds, floral buds and buds to uncover novel genes involved in regulating the phase transition from vegetative to reproductive growth. Next, we identified important regulatory genes involved in the photoperiod pathway and the control of flower organ identification to create a list of candidate genes for studying flowering-time regulation in chrysanthemum. These results will be helpful for elucidating the molecular mechanisms of flower development and will contribute to the development of techniques for studying flowering-time regulation, breeding and molecular biology in chrysanthemum.

## Results

### Illumina sequencing and assembly

The cDNA libraries of vegetative buds, floral buds and buds were sequenced using the Illumina HiSeq 2000 system. After stringent quality checks and data cleaning, 58,530,798, 41,424,050 and 54,035,520 clean reads were left for vegetative buds, floral buds and buds, respectively. The average proportion of clean reads for these libraries was approximately 93%.

A total of 123,354 contigs were assembled based on the high-quality reads, with a total size of 67,027,473 bp, an N50 of 641 bp and an average contig length of 543 bp. Then, scaffolds were constructed between the contigs via the paired-end relationships between the reads. A total of 95,280 scaffolds were obtained, with an N50 of 818 bp and an average length of 721 bp. We filled the intra-scaffold gaps and constructed a non-redundant unigene set from all three of the assembled datasets using CAP3 software. Finally, a total of 91,367 high-quality unigene sequences with lengths greater than 400 bp were obtained, with an average unigene length of 739 bp (Table 1).

**Table 1:** Summary for the chrysanthemum transcriptome.

Statistics	Counts	Total length (bp)	N25 (bp)	N50 (bp)	N75 (bp)	Average length (bp)	Longest (bp)	N%	GC%	Annotation counts	Annotation ratio
Contigs	123,354	67,027,473	1,153	641	411	543	13,557	2.4	37.2		
Primary uniGene	95,280	68,665,713	1,495	818	491	721	13,769	2.4	37.2		
Final unigene	91,367	67,529,658	1,543	854	503	739	13,769	2.52	37.24	43,137	47.21%

### Gene annotation and functional classification

Of the 91,367 unigenes, 43,137 (47.21% of the total) were aligned to the Nr protein (date 2014.03) and Swissprot protein databases (date 2014.03) using an E-value threshold of < 1e-5, which meant that 48,230 unigenes (52.79% of the total) had no Swiss-Prot annotations because of missing chrysanthemum genome and expressed sequence tag (EST) information (Table 1).

**Fig 1:**
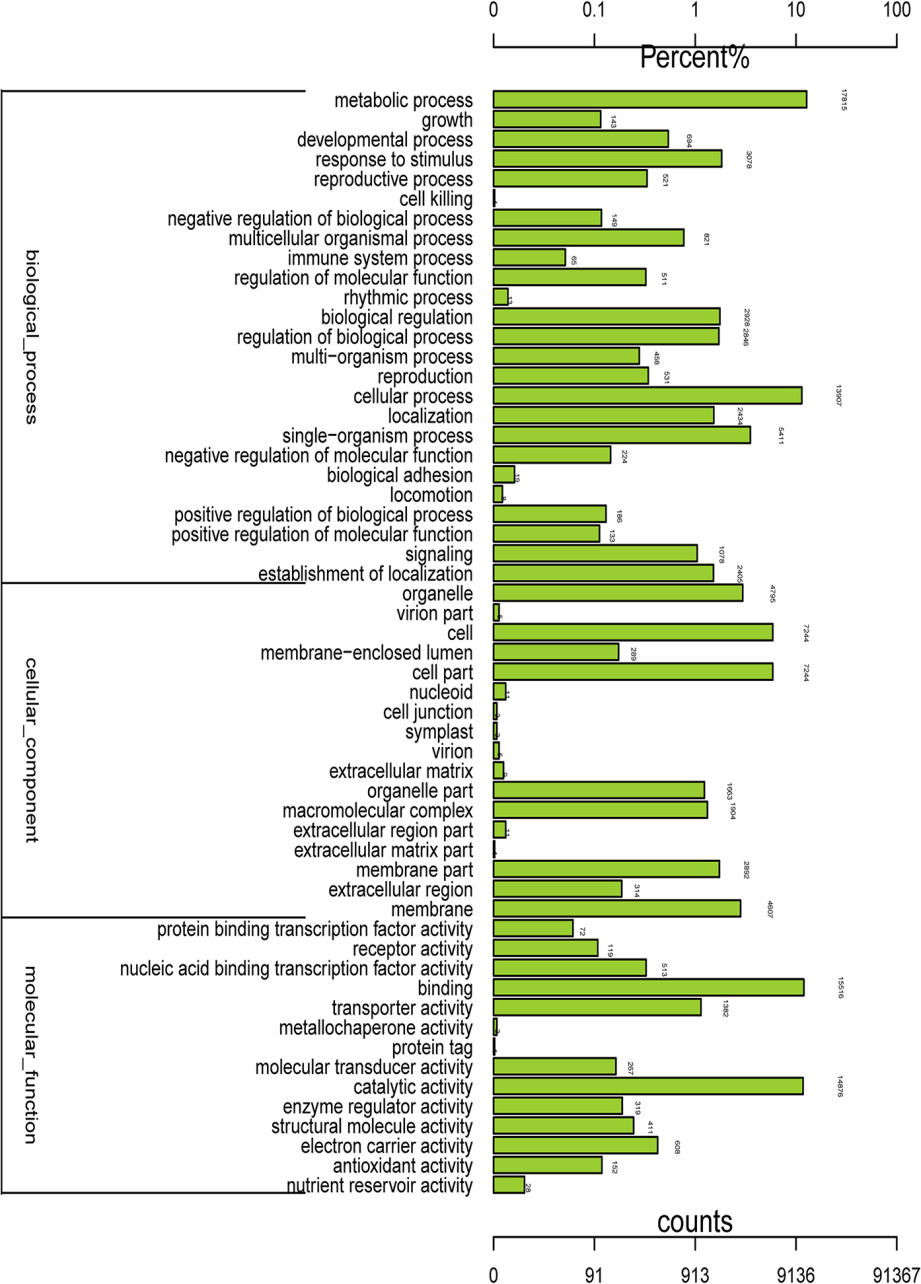
Histogram presentation of Gene Ontology classifications. The results are summarized in three main categories: biological processes, cellular components, and molecular functions. The y-axis on the left side indicates the percentage of genes in a category, and the y-axis on the right side shows the number of genes.

The international standardized gene functional classification system Gene Ontology (GO) provided three ontologies—molecular functions, cellular components and biological processes—that were useful for gene annotation and analysis. Based on the Nr annotation and Swissprot protein databases, 25,424 unigenes were classified into 56 functional GO categories using Blast2GO software [13]. A total of 19,161 unigenes were classified into 25 biochemical process categories, 10,063 unigenes were classified into 17 cellular component categories, and 22,539 unigenes were classified into 14 molecular function categories (Fig 1). For each of the three main GO classifications, the ‘metabolic process’ (in ‘biological process’), ‘cell part’ (in ‘cell component’), and ‘binding’ (in ‘molecular function’) terms were dominant in level 2. And 17,815 (92.98%), 7244 (71.98%), and 15,516 (68.84%) unigenes were assigned to ‘metabolic process’ (GO:0008152), ‘cell part’ (GO:0044464) and ‘binding’ (GO:0005488), respectively. It was indicated that complex metabolic processes regulated by a wide range of genes that also interact with one another in cell parts were activated during the development of flower in chrysanthemum. In addition, we also noted high percentages of genes from the ‘cellular process’ (GO:0009987), ‘cell’ (GO:0005623), and ‘catalytic activity’ (GO:0003824) categories, which included 13,907 (72.58%), 7244 (71.98%) and 14,876 (65.99%) unigenes, respectively. By contrast, there were relatively few genes from the ‘cell killing’ (GO:0001906), ‘extracellular matrix part’ (GO:0044420), and ‘protein tag’ (GO:0031386) categories in each of the three main GO classifications (Fig 1).

Within the 'biological process' category, we identified 13 terms related to flower development in level 6, including ‘flower development’ (GO:0009908), ‘regulation of flower development’ (GO:0009909), ‘photoperiodism, flowering’ (GO:0048573), ‘positive regulation of flower development’ (GO:0009911), ‘regulation of photoperiodism, flowering’ (GO:2000028), ‘negative regulation of flower development’ (GO:0009910), ‘regulation of long-day photoperiodism, flowering’ (GO:0048586), ‘long-day photoperiodism, flowering’ (GO:0048574), ‘negative regulation of long-day photoperiodism, flowering’ (GO:0048579), ‘flower calyx development’ (GO:0048464), ‘short-day photoperiodism, flowering’ (GO:0048575), ‘flower morphogenesis’ (GO:0048439) and ‘regulation of short-day photoperiodism, flowering’ (GO:0048587). In total, 113 unigenes were assigned to these 13 terms, of which 86 were annotated as uncharacterized or predicted proteins and the remainder showing homology to F-box family genes and *GIGANTEA* etc. (S1 Table).

**Fig 2:**
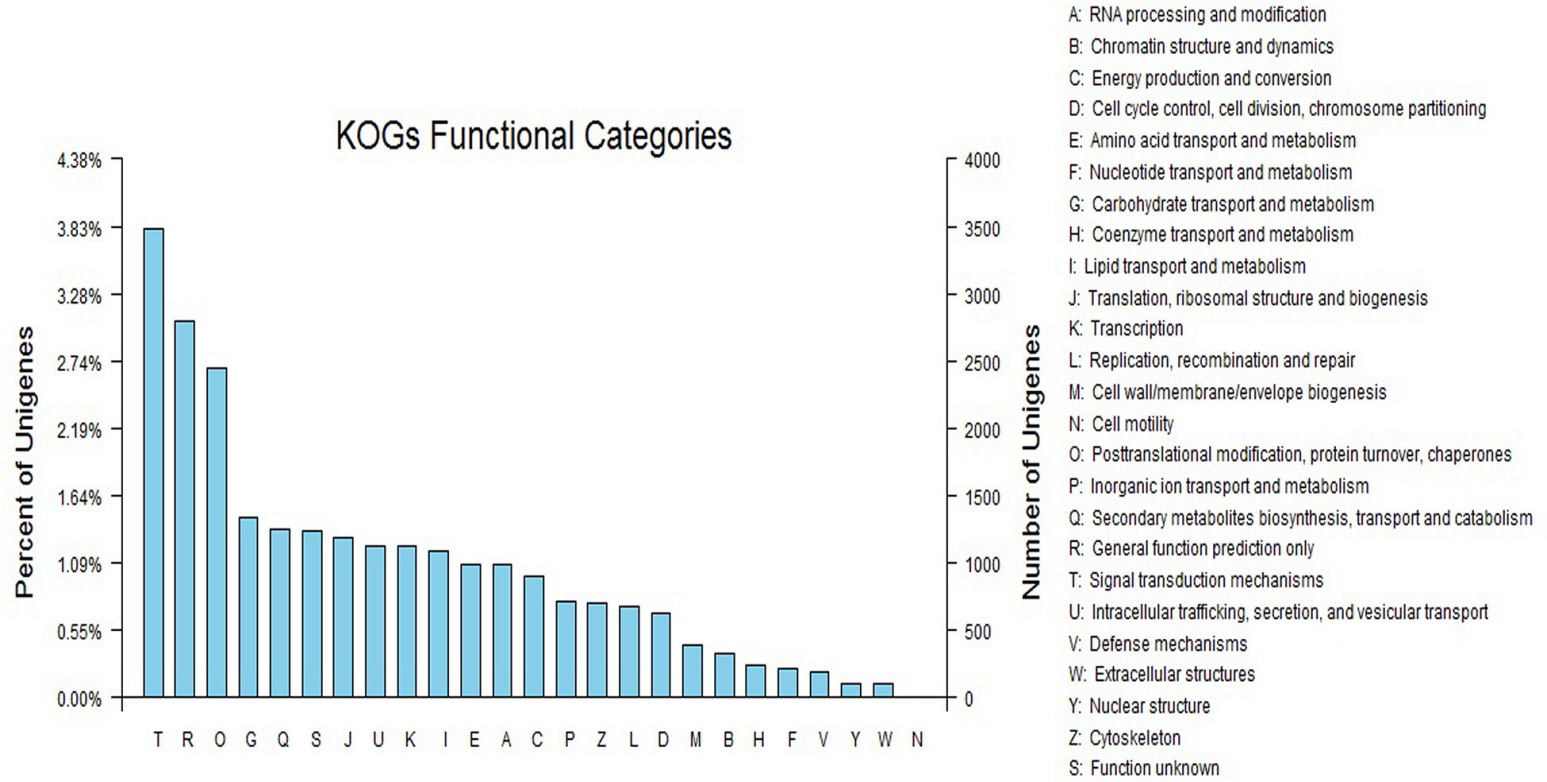
EuKaryotic Orthologous Groups (KOG) classifications in chrysanthemum. A total of 24,321 sequences with KOG classifications within the 25 categories are shown.

As a eukaryote-specific version of the Clusters of Orthologous Groups (COG) tool, euKaryotic Orthologous Groups (KOG) is used to identify orthologous and paralogous proteins, providing a way to identify Joint Genome Institute (JGI)-predicted genes by KOG classification or ID. The annotated sequences were further searched for genes involved in KOG classifications for evaluating the completeness of our transcriptome library and the effectiveness of our annotation process. Of 43,137 Nr hits, 24,321 sequences were assigned to KOG classifications. Among the 25 KOG categories, the cluster for ‘Signal transduction mechanisms’ (3487, 14.34%) represented the largest group, followed by ‘General function prediction only’ (2798, 11.50%) and ‘Posttranslational modification, protein turnover, chaperones’ (2453, 10.09%). The ‘Nuclear structure’ (108, 0.44%), ‘Extracellular structures’ (104, 0.43%), and ‘Cell motility’ (8, 0.032%) categories represented the smallest groups (Fig 2).

To further analyze the chrysanthemum transcriptome, all unigenes were compared with the Kyoto Encyclopedia of Genes and Genomes (KEGG) database using BLASTx with an E-value threshold of < 1e-5. Of the 43,137 unigenes, 13,704 had significant matches in the database and were assigned to 285 KEGG pathways (Table 2). The most represented pathways were ‘Metabolic pathways’ (3426 members), ‘Biosynthesis of secondary metabolites’ (1881 members), ‘Microbial metabolism in diverse environments’ (755 members), ‘Biosynthesis of amino acids’ (473 members), and ‘Carbon metabolism’ (463 members). In addition, 372 unigenes were assigned to ‘plant hormone signal transduction’. The annotations provide a valuable resource to research the processes, functions, and pathways involved in floret development in chrysanthemum.

**Table 2:** Categorization of Chrysanthemum unigenes to KEGG biochemical pathways.

KEGG Categories	Mapped ko	Unigene NO.	Rotio of NO.	Parthway ID
Metabolic pathways	744	3426	25.00%	ko01100
Biosynthesis of secondary metabolites	348	1881	13.73%	ko01110
Microbial metabolism in diverse environments	128	755	5.51%	ko01120
Biosynthesis of amino acids	97	473	3.45%	ko01230
Carbon metabolism	84	463	3.38%	ko01200
Starch and sucrose metabolism	35	421	3.07%	ko00500
Protein processing in endoplasmic reticulum	75	401	2.93%	ko04141
Epstein-Barr virus infection	58	398	2.90%	ko05169
RNA polymerase	23	395	2.88%	ko03020
Plant hormone signal transduction	39	372	2.71%	ko04075
mRNA surveillance pathway	46	368	2.69%	ko03015
Influenza A	19	365	2.66%	ko05164
Plant-pathogen interaction	35	362	2.64%	ko04626
Isoquinoline alkaloid biosynthesis	7	353	2.58%	ko00950
Spliceosome	83	344	2.51%	ko03040
Drug metabolism—other enzymes	12	338	2.47%	ko00983
RNA transport	80	303	2.21%	ko03013
Ribosome	112	301	2.20%	ko03010
Transcriptional misregulation in cancer	8	284	2.07%	ko05202
RNA degradation	45	274	2.00%	ko03018
Purine metabolism	81	261	1.90%	ko00230
Glycolysis / Gluconeogenesis	32	257	1.88%	ko00010
Fatty acid metabolism	28	249	1.82%	ko01212
Aminoacyl-tRNA biosynthesis	25	233	1.70%	ko00970
Amino sugar and nucleotide sugar metabolism	37	232	1.69%	ko00520
Viral carcinogenesis	41	229	1.67%	ko05203
Endocytosis	35	222	1.62%	ko04144
Neurotrophin signaling pathway	12	222	1.62%	ko04722
Pyruvate metabolism	30	220	1.61%	ko00620
Ubiquitin mediated proteolysis	55	216	1.58%	ko04120
Osteoclast differentiation	4	207	1.51%	ko04380
Cell cycle	55	206	1.50%	ko04110
Phenylpropanoid biosynthesis	18	201	1.47%	ko00940
Protein export	24	195	1.42%	ko03060
Oxidative phosphorylation	71	194	1.42%	ko00190
Toll-like receptor signaling pathway	5	189	1.38%	ko04620
Pertussis	5	187	1.36%	ko05133
Apoptosis	6	183	1.34%	ko04210
NF-kappa B signaling pathway	6	181	1.32%	ko04064

### Comparison of transcriptomes between vegetative buds, floral buds and buds

#### The common set of unigenes between vegetative buds, floral buds and buds

**Fig 3:**
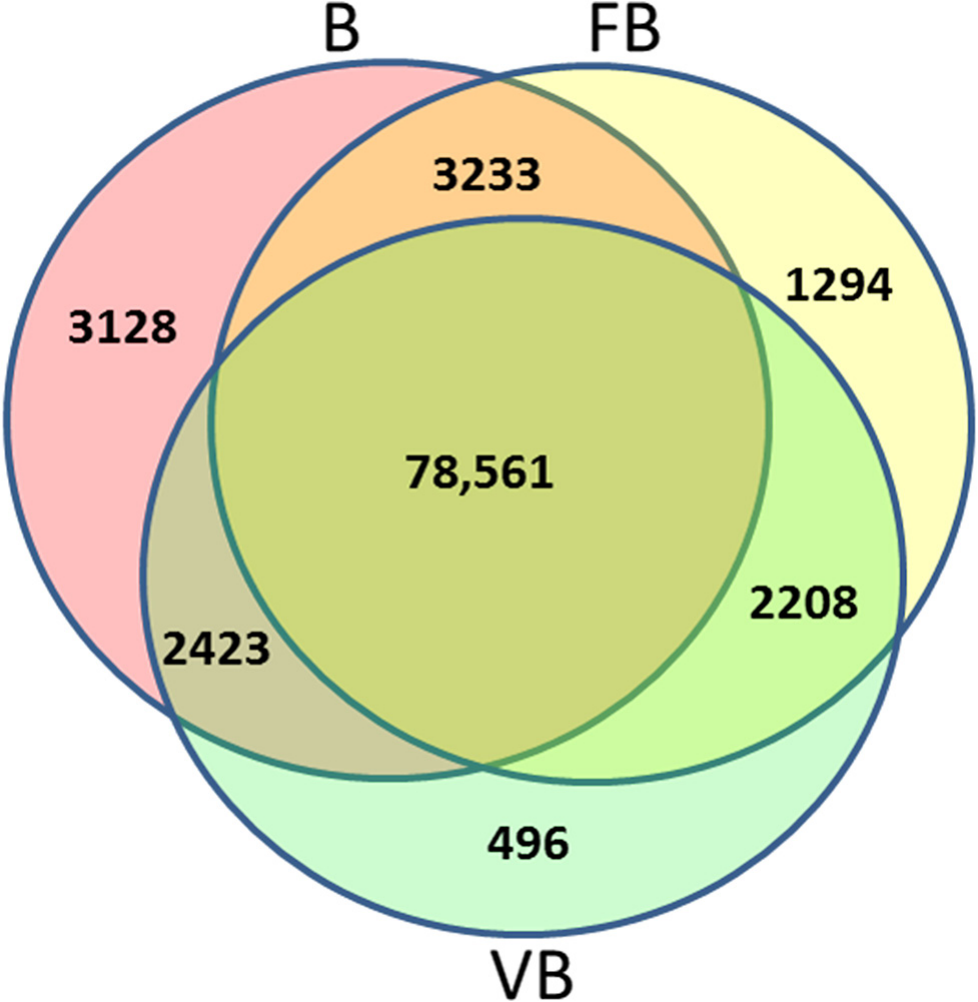
Venn diagram of the number of unigenes with reads per kilobases per million mapped (RPKM) > 0.3 in vegetative buds (VB), floral buds (FB) and buds (B).

The number of unigenes with an RPKM value of > 0.3 that were shared by vegetative buds and floral buds, floral buds and buds, and vegetative buds and buds were 80,769, 81,794, and 80,984, respectively. Vegetative buds, floral buds and buds shared 78,561 unigenes in common (Fig 3). By contrast, 496, 1294 and 3128 unigenes showed specific expression in vegetative buds, floral buds and buds, respectively. During development from vegetative growth to flower differentiation and bud formation, an increasing number of genes are expressed in chrysanthemum.

#### DEGs between vegetative buds, floral buds and buds

**Fig 4:**
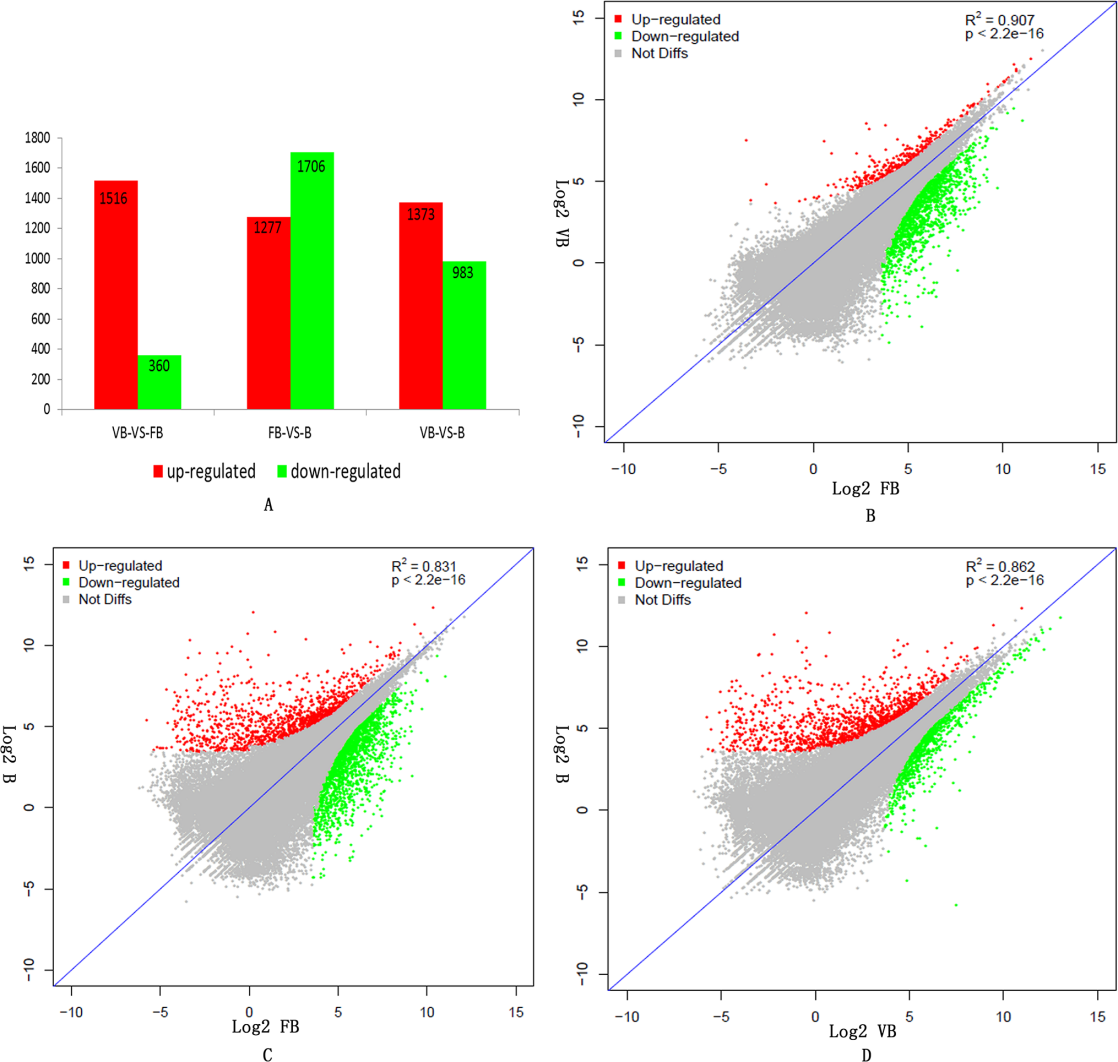
Gene expression differences among the different samples. A. The numbers of up- and down-regulated genes between vegetative buds (VB), floral buds (FB) and buds (B). B. The correlation scatter plot of gene expression differences between vegetative buds (VB) and floral buds (FB). C. The correlation scatter plot of gene expression differences between floral buds (FB) and buds (B). D. The correlation scatter plot of gene expression differences between buds (B) and vegetative buds (VB).

The resulting reads were mapped to our reference transcriptome; statistical significance values were taken to be reliable with an RPKM value ≥ 2 in at least one of the three samples. Transcriptomes were compared, and differentially expressed genes (DEGs) were analyzed in three samples: vegetative buds, floral buds and buds. To determine which of the unigenes were differentially expressed, we filtered with an FDR ≤ 0.05 and a |log2 (ratio)| ≥ 2; 1,876 DEGs were significantly changed in expression between vegetative buds and floral buds, of which 1,516 DEGs were up-regulated and 360 DEGs were down-regulated in the floral buds (Fig 4A).

GO and KEGG Pathway enrichment analyses were performed on the DEGs to identify differences in the biological processes and pathways between vegetative buds and floral buds. In total, 998 DEGs were enriched in GO categories. Table 3 listed some representative enriched GO terms between vegetative buds and floral buds. In the 'biological process' category, the dominant terms were the following: ‘protein phosphorylation’ (GO:0006468), ‘oxidation-reduction process’ (GO:0055114), ‘regulation of transcription, DNA-dependent’ (GO:0006355), ‘phosphorylation’ (GO:0016310), ‘metabolic process’ (GO:0008152), ‘transcription, DNA-dependent’ (GO:0006351), ‘carbohydrate metabolic process’ (GO:0005975) and ‘defense response’ (GO:0006952). In the 'cellular component' category, the most representative terms were the following: ‘membrane’ (GO:0016020), ‘integral to membrane’ (GO:0016021), ‘nucleus’ (GO:0005634), ‘plasma membrane’ (GO:0005886). Finally, in the 'molecular function' category the most representative terms were the following: ‘ATP binding’ (GO:0005524), ‘transferase activity, transferring phosphorus-containing groups’ (GO:0016772), and ‘protein kinase activity’ (GO:0004672). As shown in Table 4, for the KEGG pathway analysis, the dominant pathways were the following: ‘Metabolic pathways’ (ko01100), ‘Biosynthesis of secondary metabolites’ (ko01110), ‘RNA polymerase’ (ko03020), ‘Isoquinoline alkaloid biosynthesis’ (ko00950), and ‘Plant hormone signal transduction’ (ko04075). Interestingly, for many of the enriched GO terms, the majority of or all DEGs were up-regulated in floral buds relative to vegetative buds, as was the case for ‘phosphorylation’ (GO:0016310), ‘carbohydrate metabolic process’ (GO:0005975), ‘defense response’ (GO:0006952), ‘response to stress’ (GO:0006950), and ‘signal transduction’ (GO:0007165), among others. Similarly, most or all of the DEGs in many KEGG pathways were up-regulated in floral buds relative to vegetative buds, as was the case for ‘RNA polymerase’ (ko03020), ‘Isoquinoline alkaloid biosynthesis’ (ko00950), and ‘Starch and sucrose metabolism’ (ko00500), among others. Detailed information on the DEGs that were differentially expressed between vegetative buds and floral buds is listed in S2 and S3 Tables.

**Table 3:** GO enrichment of DEGs between floral buds and vegetative buds.

GO ID	GO term	Type	NO. of DEGs in the GO term	NO. of down-regulated genes in floral buds	NO. of up-regulated genes in floral buds	NO. of DEGs in all GO terms
GO:0005524	ATP binding	Molecular function	210	23	187	998
GO:0016772	transferase activity, transferring phosphorus-containing groups	Molecular function	145	12	133	998
GO:0006468	protein phosphorylation	Biological process	142	11	131	998
GO:0004672	protein kinase activity	Molecular function	142	11	131	998
GO:0000166	nucleotide binding	Molecular function	142	27	115	998
GO:0004674	protein serine/threonine kinase activity	Molecular function	108	2	106	998
GO:0046872	metal ion binding	Molecular function	129	31	98	998
GO:0055114	oxidation-reduction process	Biological process	142	46	96	998
GO:0016491	oxidoreductase activity	Molecular function	131	43	88	998
GO:0016020	membrane	Cellular component	112	26	86	998
GO:0016021	integral to membrane	Cellular component	94	16	78	998
GO:0006355	regulation of transcription, DNA-dependent	Biological process	91	16	75	998
GO:0016787	hydrolase activity	Molecular function	81	8	73	998
GO:0003824	catalytic activity	Molecular function	86	15	71	998
GO:0016740	transferase activity	Molecular function	94	27	67	998
GO:0003677	DNA binding	Molecular function	96	30	66	998
GO:0008152	metabolic process	Biological process	97	36	61	998
GO:0003700	sequence-specific DNA binding transcription factor activity	Molecular function	64	8	56	998
GO:0016310	phosphorylation	Biological process	57	2	55	998
GO:0005634	nucleus	Cellular component	86	31	55	998
GO:0016301	kinase activity	Molecular function	57	2	55	998
GO:0008270	zinc ion binding	Molecular function	58	13	45	998
GO:0006351	transcription, DNA-dependent	Biological process	46	7	39	998
GO:0005975	carbohydrate metabolic process	Biological process	38	1	37	998
GO:0006952	defense response	Biological process	34	1	33	998
GO:0004553	hydrolase activity, hydrolyzing O-glycosyl compounds	Molecular function	34	1	33	998
GO:0043531	ADP binding	Molecular function	32	0	32	998
GO:0005506	iron ion binding	Molecular function	43	13	30	998
GO:0005509	calcium ion binding	Molecular function	31	1	30	998
GO:0020037	heme binding	Molecular function	38	11	27	998
GO:0009055	electron carrier activity	Molecular function	38	11	27	998
GO:0006950	response to stress	Biological process	26	0	26	998
GO:0005886	plasma membrane	Cellular component	30	4	26	998
GO:0007165	signal transduction	Biological process	24	0	24	998

**Table 4:** KEGG enrichment of DEGs between floral buds and vegetative buds.

Pathway ID	KEGG name	NO. of DEGs in the pathway	NO. of down-regulated genes in floral buds in the pathway	NO. of up-regulated genes in floral buds in the pathway	NO. of DEGs in all pathways
ko01100	Metabolic pathways	173	62	111	406
ko01110	Biosynthesis of secondary metabolites	112	45	67	406
ko03020	RNA polymerase	41	2	39	406
ko00950	Isoquinoline alkaloid biosynthesis	35	0	35	406
ko04075	Plant hormone signal transduction	29	7	22	406
ko00983	Drug metabolism—other enzymes	28	0	28	406
ko05202	Transcriptional misregulation in cancer	27	5	22	406
ko04626	Plant-pathogen interaction	27	0	27	406
ko05164	Influenza A	26	0	26	406
ko01120	Microbial metabolism in diverse environments	26	14	12	406
ko00500	Starch and sucrose metabolism	24	0	24	406
ko05133	Pertussis	23	0	23	406
ko00940	Phenylpropanoid biosynthesis	23	9	14	406
ko04722	Neurotrophin signaling pathway	23	0	23	406
ko00945	Stilbenoid, diarylheptanoid and gingerol biosynthesis	22	11	11	406
ko04210	Apoptosis	22	0	22	406
ko04620	Toll-like receptor signaling pathway	22	0	22	406
ko04380	Osteoclast differentiation	22	0	22	406
ko04064	NF-kappa B signaling pathway	21	0	21	406
ko04141	Protein processing in endoplasmic reticulum	20	0	20	406

A total of 2,983 DEGs (1,706 down-regulated and 1,277 up-regulated in buds) were identified between floral buds and buds (Fig 4A). In total, 1712 DEGs between floral buds and buds were enriched in GO categories. As shown in Table 5, GO enrichment analysis of down-regulated genes in the 'biological processes' category identified a number of categories, including ‘protein phosphorylation’ (GO:0006468), ‘oxidation-reduction process’ (GO:0055114), ‘metabolic process’ (GO:0008152) and ‘regulation of transcription, DNA-dependent’ (GO:0006355). For the 'molecular function' category, the dominant terms among the down-regulated genes were ‘ATP binding’ (GO:0005524), ‘transferase activity, transferring phosphorus-containing groups’ (GO:0016772) and ‘protein kinase activity’ (GO:0004672). For the 'cellular component' category, the dominant terms among the down-regulated genes were ‘membrane’ (GO:0016020), ‘integral to membrane’ (GO:0016021) and ‘nucleus’ (GO:0005634). As shown in Table 5, GO enrichment analysis of the up-regulated genes in buds relative to floral buds identified genes involved in ‘metabolic process’ (GO:0008152, in biological processes), ‘hydrolase activity’ (GO:0016787, in molecular function), and ‘membrane’ (GO:0016020, in cellular component). In total, 546 DEGs were enriched for KEGG pathways, and the dominant pathways were ‘Metabolic pathways’ (ko01100), ‘Biosynthesis of secondary metabolites’ (ko01110) and ‘Isoquinoline alkaloid biosynthesis’ (ko00950). Detailed information on the DEGs between floral buds and buds is listed in S4 and S5 Tables.

**Table 5:** GO enrichment of DEGs between buds and floral buds.

GO ID	GO term	Type	NO. of DEGs in the GO term	NO. of down-regulated genes in buds	NO. of up-regulated genes in buds	NO. of DEGs in all GO terms
GO:0005524	ATP binding	Molecular function	309	264	45	1712
GO:0016772	transferase activity, transferring phosphorus-containing groups	Molecular function	201	182	19	1712
GO:0004672	protein kinase activity	Molecular function	194	179	15	1712
GO:0006468	protein phosphorylation	Biological process	194	179	15	1712
GO:0000166	nucleotide binding	Molecular function	251	165	86	1712
GO:0004674	protein serine/threonine kinase activity	Molecular function	165	153	12	1712
GO:0016020	membrane	Cellular component	243	138	105	1712
GO:0055114	oxidation-reduction process	Biological process	236	111	125	1712
GO:0046872	metal ion binding	Molecular function	196	110	86	1712
GO:0016491	oxidoreductase activity	Molecular function	210	107	103	1712
GO:0016021	integral to membrane	Cellular component	187	106	81	1712
GO:0016787	hydrolase activity	Molecular function	199	89	110	1712
GO:0016740	transferase activity	Molecular function	139	86	53	1712
GO:0008152	metabolic process	Biological process	214	85	129	1712
GO:0006355	regulation of transcription, DNA-dependent	Biological process	106	85	21	1712
GO:0006952	defense response	Biological process	92	85	7	1712
GO:0043531	ADP binding	Molecular function	85	85	0	1712
GO:0003824	catalytic activity	Molecular function	162	82	80	1712
GO:0003677	DNA binding	Molecular function	111	79	32	1712
GO:0005634	nucleus	Cellular component	103	73	30	1712
GO:0003700	sequence-specific DNA binding transcription factor activity	Molecular function	84	71	13	1712
GO:0016301	kinase activity	Molecular function	82	71	11	1712
GO:0016310	phosphorylation	Biological process	82	71	11	1712
GO:0007165	signal transduction	Biological process	65	63	2	1712

A total of 2,356 DEGs (983 down-regulated and 1,373 up-regulated in buds) were identified between buds and vegetative buds (Fig 4A). In addition, we also identified 174 DEGs specifically expressed in buds relative to floral buds, which contained many genes involved in pistil and stamen development (S6 Table).

To identify unigenes with significant changes in expression, the correlations in gene expression between the three samples were studied using an algorithm developed from the correlation scatter plot. As shown in Fig 4B, the difference in gene expression was smallest between vegetative buds and floral buds, yielding a strong correlation in gene expression (R^2^ = 0.907). By contrast, the difference in gene expression was the largest between floral buds and buds, leading to a poor correlation in gene expression (R^2^ = 0.831, Fig 4C).

**Fig 5:**
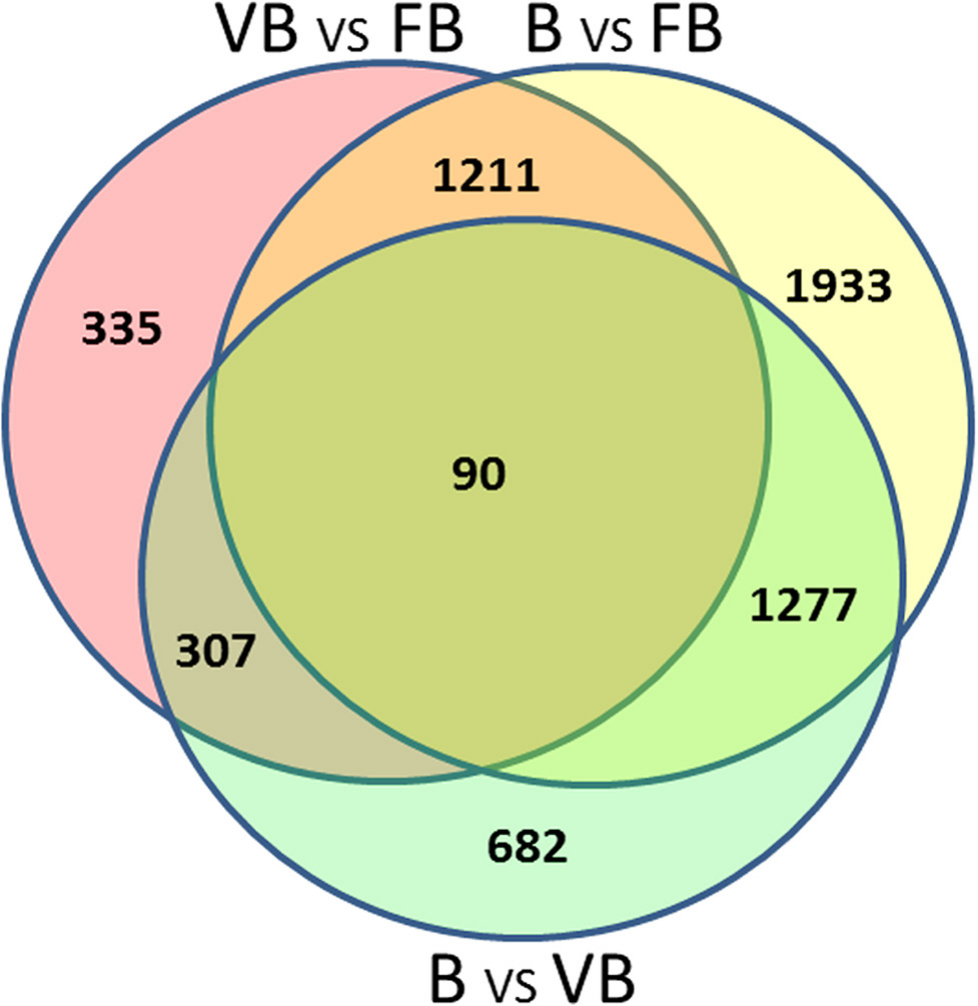
Venn diagram of the number of DEGs between vegetative buds (VB), floral buds (FB) and buds (B).

Among the samples from three different developmental periods in chrysanthemum, the number of up-regulated genes in floral buds relative to vegetative buds (1516) was greater than the number in buds relative to floral buds (1277). Fewer genes (360) were down-regulated in floral buds relative to vegetative buds than the number down-regulated (1706) in buds relative to floral buds. Indeed, there were only 90 common DEGs among vegetative buds, floral buds and buds (Fig 5). In total, 1301 DEGs in vegetative buds and floral buds were also differentially expressed in floral buds and buds. By contrast, 397 DEGs in vegetative buds and floral buds were also differentially expressed in buds and vegetative buds (Fig 5). We also found that 1367 DEGs in floral buds and buds were differentially expressed in buds and vegetative buds (Fig 5).

#### Up-regulated unigenes during flower differentiation

By comparing the transcriptomes of vegetative buds, floral buds and buds, we found that many unigenes were dramatically up-regulated in floral buds and down-regulated in both vegetative buds and buds, suggesting that these genes play an important role during flower differentiation.

**Fig 6:**
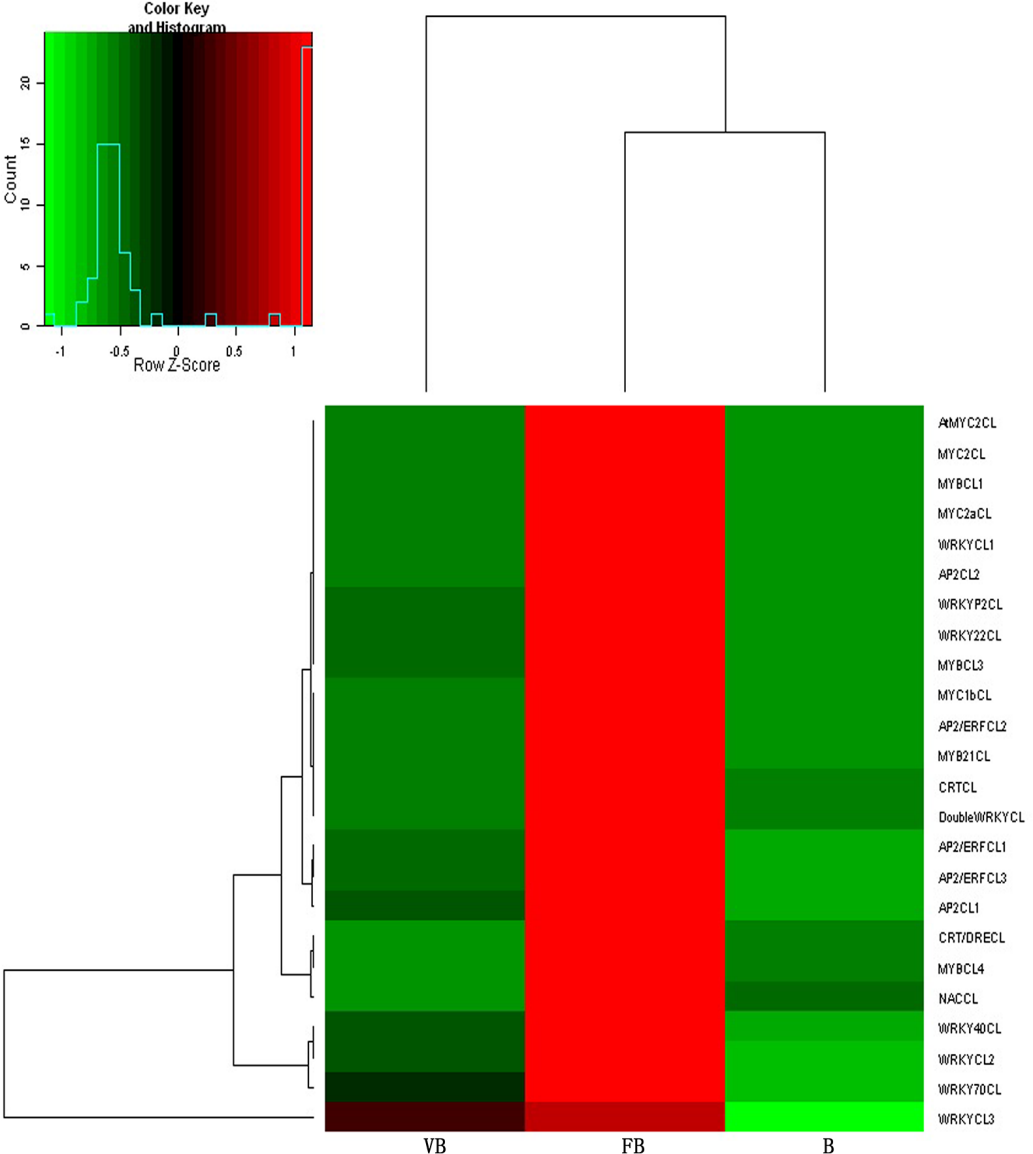
A heat-map showing transcription factor family genes that were differentially expressed between vegetative buds (VB), floral buds (FB) and buds (B) in chrysanthemum. Columns and rows in the heat maps represent samples and genes, respectively. Sample names are displayed below the heat maps. The color scale indicates fold-change in gene expression. Red indicates high expression and green indicates low expression.

The expression levels of many important transcription factor genes, including the homologs of AP2, MYB, MYC, WRKY, NAC and CRT, were dramatically up-regulated in floral buds (Table 6 and Fig 6). Interestingly, we identified one CRT-binding gene (*CRTCL*) that was highly expressed in floral buds, weakly expressed in vegetative buds, and not expressed at all in buds (Table 6). One homolog of AP2 (*AP2CL3*) was assigned to “organ morphogenesis” (GO:0009887) under the 'biological process' category. Furthermore, many other homologs of these transcription factors were assigned to the ‘nucleus’ (GO:0005634), ‘DNA binding’ (GO:0003677) and ‘regulation of transcription, DNA-dependent’ (GO:0006355) categories under 'cellular component,' 'molecular function' and 'biological process,' respectively.

**Table 6:** The transcription factors family genes up-regulated in floral buds.

Gene Name	Protein description	Vegetative buds (RPKM)	Floral buds (RPKM)	Buds (RPKM)	Fold change (Floral buds/Vegetative buds)	Fold change (Floral Buds/Buds)	P-value	FDR
*AP2/ERFCL2*	AP2/ERF domain-containing transcription factor	2.128	18.600854	1.552713	8.742122	11.97958	2.22E-05	7.50E-05
*AP2/ERFCL3*	AP2/ERF and B3 domain-containing transcription factor	5.11	31.521888	0.399682	6.168944	78.86742	8.01E-09	6.22E-07
*AP2CL2*	AP2 domain-containing transcription factor	3.32	24.286505	1.709164	7.315126	14.20958	5.68E-06	2.74E-04
*AP2CL1*	AP2 domain class transcription factor	28.32	66.328704	19.98047	2.342184	3.319677	4.42E-07	2.62E-05
*AP2/ERFCL1*	AP2/ERF transcription factor	39.06	206.89651	12.35639	5.296929	16.74409	5.99E-46	3.80E-43
*WRKYCL1*	WRKY transcription factor	5.025	53.435992	1.397138	10.63388	38.24674	4.58E-14	6.16E-12
*WRKYCL2*	WRKY transcription factor	20.95	79.003856	2.02556	3.771188	39.00346	2.12E-20	4.66E-18
*WRKY22CL*	WRKY22 transcription factor	2.088	16.306073	0.56334	7.811082	28.94536	1.45E-04	5.15E-03
*WRKYP2CL*	WRKYP2 transcription factor	5.057	19.878	3.570632	3.930575	5.567082	1.55E-03	4.02E-02
*DoubleWRKYCL*	Double WRKY type transfactor	2.455	43.658545	2.047324	17.78694	21.32469	2.51E-10	2.32E-08
*WRKY70CL*	WRKY transcription factor	35.8	100.88176	3.974785	2.818084	25.38043	2.74E-25	7.82E-23
*WRKY40CL*	WRKY transcription factor	5.058	20.196108	0.53264	3.993031	37.91703	1.10E-05	5.01E-04
*WRKYCL3*	WRKY transcription factor	16.02	21.976893	0.56679	1.372259	38.77428	5.75E-06	2.76E-04
*MYBCL1*	Myb-like protein	9.508	165.33942	0.926526	17.3897	178.451	2.43E-48	1.70E-45
*MYB21CL*	MYB21 transcription factor	3.314	50.074228	1.290033	15.11119	38.81625	3.27E-13	4.11E-11
*MYBCL3*	Myb-related transcription factor	18.27	74.406767	13.14954	4.071955	5.658508	3.15E-11	3.24E-09
*MYBCL4*	Myb-like DNA-binding protein	2.794	24.532345	3.943807	8.780541	6.220473	1.04E-04	3.83E-03
*MYC2CL*	MYC2 transcription factor	27.05	185.92307	17.2481	6.87242	10.77934	3.17E-36	1.41E-33
*MYC2aCL*	MYC2a transcription factor	36.76	223.89981	22.79817	6.091271	9.820956	2.19E-42	1.26E-39
*MYC1bCL*	MYC1b transcription factor	27.82	208.84963	22.00965	7.508086	9.489001	1.51E-38	7.33E-36
*AtMYC2CL*	Transcription factor	19.19	127.7421	12.04762	6.656098	10.6031	7.34E-25	2.03E-22
*NACCL*	NAC transcription factor	0.629	33.870427	4.568384	53.83805	7.414094	2.45E-06	1.29E-04
*CRT/DRECL*	CRT/DRE binding factor	1.785	170.54517	7.639998	95.52265	22.32267	3.44E-48	2.40E-45
*CRTCL*	CRT binding factor	0.05	12.4159	0	248.318		1.83E-03	4.63E-03

The genes that were up-regulated during flower differentiation also included many genes that are responsive to phytohormones or are involved in phytohormone synthesis, as well as sugar synthesis/transport genes, kinase-like genes, ubiquitin-like genes, and resistance genes; numerous uncharacterized protein coding genes were also identified. These genes were enriched for the ‘carbohydrate metabolic process’ (GO:0005975), ‘signal transduction’ (GO:0007165), ‘protein kinase activity’ (GO:0004672) and ‘defense response’ (GO:0006952) categories and are listed in S7–S10 Tables.

### Identification of genes involved in the photoperiod pathway in chrysanthemum

**Fig 7:**
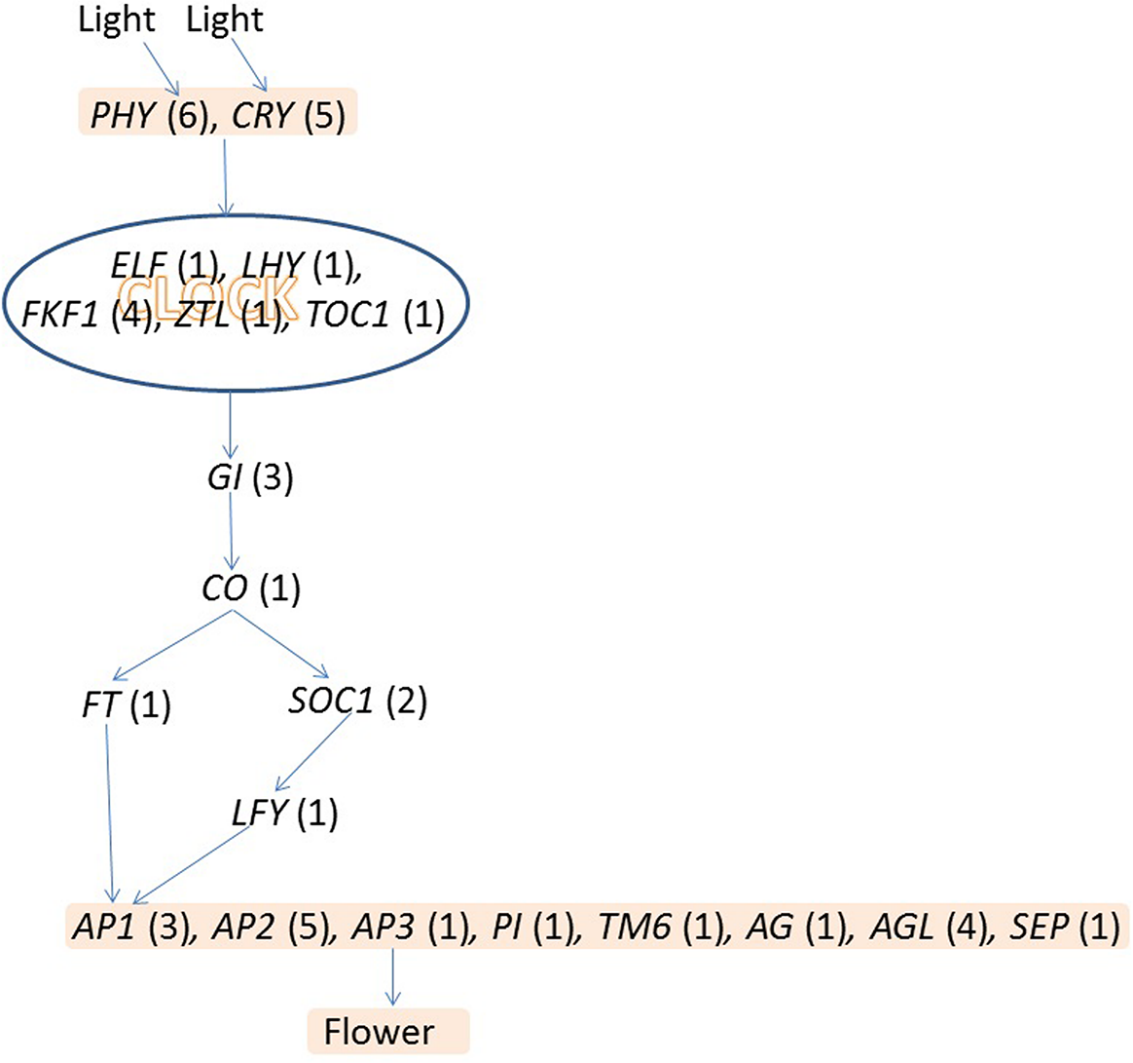
A schematic of flowering regulatory networks in the photoperiod pathway of *Chrysanthemum morifolium*. Arrows indicate activation. All homologs of the regulators involved in the photoperiod pathway are listed in S11 Table. Numbers represent the members of the corresponding genes identified in the chrysanthemum transcriptome.

Chrysanthemum is a typical short-day plant; it can flower in response to a single short day. We searched for homologous genes of the important regulators involved in the photoperiod pathway in chrysanthemum. In Arabidopsis, many genes required for the daylength response have been identified using molecular-genetic approaches, among which some members encode regulatory proteins specifically playing roles in the regulation of flowering; others encode components in light signal transduction pathways or pathways related to circadian signaling, including *CRYPTOCHROME* (*CRY*), *PHYTOCHROME* (*PHY*), *LATE ELONGATED HYPOCOTYL* (*LHY*), *EARLY FLOWERING 4* (*EFL4*), *FLAVIN-BINDING KELCH REPEAT F-BOX 1* (*FKF1*), *TIMING OF CAB 1* (*TOC1*), *ZEITLUPE* (*ZTL*) and *GIGANTEA* (*GI*) and others [3]. In this study, many unigenes from the chrysanthemum transcriptome were identified as homologous genes of the photoreceptor and circadian clock components in the photoperiod pathway (Fig 7). Based on the protein annotations of the chrysanthemum transcriptome sequences, a number of genes were identified, including five homologs of *CRY* (*CRYCL1*, *CRYCL2*, *CRYCL3*, *CRYCL4*, *CRYCL5*), six homologs of *PHY* (*PHYCL1*, *PHYCL2*, *PHYCL3*, *PHYCL4*, *PHYCL5*, *PHYCL6*), one homolog of *LHY* (*LHYCL*), one homolog of *EFL4* (*EFL4CL*), four homologs of *FKF1* (*FKF1CL1*, *FKF1CL2*, *FKF1CL3*, *FKF1CL4*), one homolog of *TOC1* (*TOC1CL*), one homolog of *ZTL* (*ZTLCL*), and three homologs of *GI* (*GICL1*, *GICL2*, *GICL3*). *CONSTANS* (*CO*) is a key regulator of the photoperiod response, and one homolog of *CO* (*COCL1*) was identified. *FT* (*Flowering Locus T*) is the early target of *CO*, and one homolog of *FT* (*FTCL*) was also identified (Fig 7). In the photoperiod pathway, many MADS box genes also play key roles in promoting floral meristem identity, including *SUPPRESSOR OF CONSTANS1* (*SOC1*), *SHORT VEGETATIVE PHASE* (*SVP*), *LEAFY* (*LFY*), *APETALA1* (*AP1*), *GIGANTEA* (*GI*), *AGAMOUS* (*AG*) and *PISTILLATA* (*PI*). *APETALA2* (*AP2*) also play key roles in promoting floral meristem identity. And homologs of these MADS box genes and *AP2* were also identified (Fig 7). *LEAFY* (*LFY*) is a vital regulator of the specification of floral meristem identity. This gene is initially expressed very early throughout the presumptive floral meristem, and its expression leads to a cascade of transcriptional activities that control floral meristem development and meristem identity [4]. We found one homolog of *LFY* (*LFYCL*) that was significantly up-regulated in floral buds. SOC1 is an upstream regulatory gene of *LFY*. Two homologs of *SOC1*, *SOC1CL1* and *SOC1CL2*, were identified in chrysanthemum, and their expression levels were significantly up-regulated in the floral bud stage. *AP1* is ‘A-class’ like gene; *LFY* directly activates *AP1* transcription [4, 14].

In *Arabidopsis*, *AP1* and *LFY* are expressed throughout the floral meristem and are thought to regulate floral initiation via cell-to-cell signaling [15]. *AP1CL1* and *AP1CL2*, which are homologous to *AP1*, were initially expressed in floral buds and exhibited higher expression levels in buds in chrysanthemum. The third homolog of *AP1*, *AP1CL3*, exhibited higher expression in vegetative buds, suggesting that *AP1CL3* is probably not involved in floral meristem identity. *AP2* is the second ‘A-class’ gene that is not a MADS-box transcription factor. Many homologs of *AP2* were identified in chrysanthemum (Fig 7), and the majority were significantly up-regulated in floral buds. The B-class genes of most core eudicot species include three different lineages: *PI*, *euAP3* and *TM6*. *TM6*-like genes appear to have been lost in *Arabidopsis* and *Antirrhinum* [16]. In chrysanthemum, we identified homologs for all three genes *PI*, *euAP3* and *TM6* (*PICL*, *AP3CL*, *TM6CL*). In addition, we identified one homolog of the C-class gene *AG* (*AGCL*) in chrysanthemum. The homologs of *AP3*, *PI*, *TM6* and *AG*, were initially expressed in the floral buds, although they showed the highest expression in buds. Annotation information for unigenes involved in the photoperiod pathway in chrysanthemum is listed in S11 Table.

### Verification of gene expression profiles by qRT-PCR

**Fig 8:**
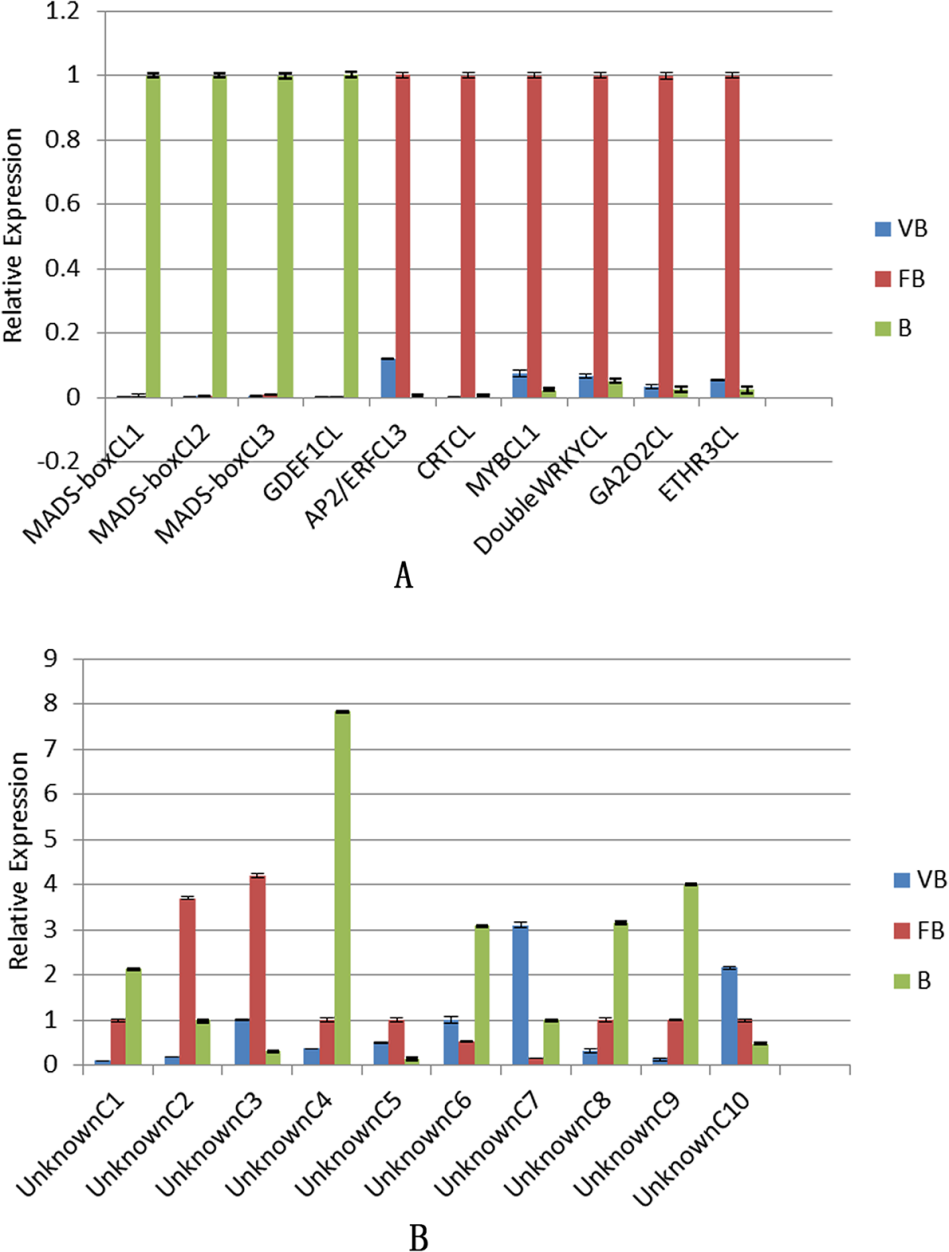
The expression profiles of 20 transcripts in *Chrysanthemum morifolium*, as measured by qRT-PCR. A. The expression profiles of 10 transcripts that were assigned as known proteins. B. The expression profiles of 10 transcripts that were assigned as uncharacterized or predicted proteins.

To further confirm the expression data of the genes in the Illumina sequencing analyses, we randomly selected 20 unigenes for qRT-PCR evaluation using the vegetative bud, floral bud and bud samples that were originally used for RNA-Seq (Fig 8). Among the 20 selected unigenes, 10 were annotated as known proteins (*MADS-boxCL1*, *MADS-boxCL2*, *MADS-boxCL3*, *GDEF1CL*, *AP2/ERFCL3*, *CRTCL*, *MYBCL1*, *DoubleWRKYCL*, *GA2O2CL*, *ETHR3CL*) and 10 were annotated as uncharacterized or predicted proteins (*UnknownC1*, *UnknownC2*, *UnknownC3*, *UnknownC4*, *UnknownC5*, *UnknownC6*, *UnknownC7*, *UnknownC8*, *UnknownC9*, *UnknownC10*). The expression of 4 genes (*GDEF1CL*, *MADS-boxCL1*, *MADS-boxCL2* and *MADS-boxCL3*) was only weakly detected in vegetative buds and floral buds by qRT-PCR (Fig 8A). Consistent with this, these genes were also assigned minimal expression levels by RNA-Seq analysis (S12 Table). The expression patterns of the 20 selected genes were nearly identical, and the gene expression levels detected by Illumina sequencing analysis were consistent with the qRT-PCR results.

## Discussion

For the chrysanthemum transcriptome, approximately 16.5 Gb of data were generated and assembled into 91,367 unigenes with an average length of 739 bp. However, only 43,137 unigenes (47.21% of the total) were annotated in the Swiss-Prot database, and the remaining 48,230 unigenes (52.79% of the total) had no Swiss-Prot annotations due to the lack of chrysanthemum genomic and EST information. The assembly unigenes in this study were assigned to an extensive range of GO categories, KEGG pathways and KOG classifications, which indicated that various transcripts are involved in chrysanthemum flower development.

Comparative analysis of the transcriptomes revealed a wealth of valuable information. As shown in Fig 3, vegetative buds, floral buds and buds shared 78,561 common unigenes, whereas only 496, 1294 and 3128 unigenes were specifically expressed in vegetative buds, floral buds and buds, respectively. Therefore, the majority of unigenes were expressed during all three developmental stages. However, during flower bud differentiation and bud development, many unigenes were up-regulated and a small number of unigenes were turned off. Many unigenes were significantly up-regulated during flower differentiation, including many important transcription factors shown in Table 6. Previous studies have shown that the AP2/ERF transcription factors are expressed in response to developmental and environmental stimuli; some also played roles downstream of the ethylene, biotic, and abiotic stress signaling pathways in model plants [17]. WRKY proteins play regulatory roles in plant defense responses [18]. MYC/MYB transcription factors are involved in the ABA-dependent pathway to up-regulate abiotic stress-responsive genes [19, 20]. NAC proteins are plant-specific transcription factors that function in diverse developmental processes and stress responses, though the biological functions of most NAC family genes remain unknown [21]. In the chrysanthemum transcriptome, AP2, WRKY, MYB, MYC, NAC genes are significantly up-regulated in floral buds relative to vegetative buds and buds, indicating that these genes play important roles in chrysanthemum flower development and should be addressed in future studies (Table 6). Interestingly, two CRT genes were identified as highly expressed in floral buds. Both CRT genes (*CRT/DRECL*, *CRTCL*) showed minimal expression in vegetative buds, and one CRT gene (*CRTCL*) was not detected in buds. It is known that CRT genes are important regulators of plant reproductive events [22]. However, our findings suggest that CRT genes likely play a vital role in regulating chrysanthemum flower bud differentiation.

Among the important transcription factors mentioned above, some are involved in the same biological processes as genes involved in phytohormone signaling, the photoperiod pathway and organ determination. For example, 27 homologous genes were assigned to ‘transcription, DNA-dependent’ (GO:0006351) in the 'biological process' category. These genes include the homologs of CRT, SOC1, SVP, AGL, AP1, AP2, AP3, PI, TM6, AG, and SEP (*CRT/DRECL*, *SOC1CL1*, *SOC1CL2*, *SVP1CL*, *SVP3CL*, *AGLCL2*, *AP1CL1*, *AP1CL2*, *AP1CL3*, *AP2/ERFCL2*, *AP2CL1*, *AP2CL2*, *AP2CL3*, *AP2*/*ERFCL1*, *AP3CL*, *PICL*, *TM6CL*, *AGCL1*, *AGCL3*, and *SEPCL*) as well as several homologous genes in the ethylene signaling pathway. Another 'biological process' term, ‘regulation of transcription, DNA-dependent’ (GO:0006355), included 37 genes that were the homologs of WRKY, NAC, and CRT (*WRKYCL1*, *WRKYCL2*, *WRKY22CL*, *DoubleWRKYCL*, *WRKY70CL*, *WRKY40CL*, *WRKYCL3*, *NACCL*, *CRT/DRECL*), LFY, SOC1, SVP, AGL, AP1, AP2, AP3, PI, TM6, AG, and SEP (*LFYCL*, *SOC1CL1*, *SOC1CL2*, *SVP1CL*, *SVP3CL*, *AGLCL2*, *AP1CL1*, *AP1CL2*, *AP1CL3*, *AP2/ERFCL2*, *AP2CL3*, *AP2CL2*, *AP2CL1*, *AP2/ERFCL1*, *AP3CL*, *TM6CL*, *PICL*, *AGCL1*, *AGCL3*, and *SEPCL*), as well as genes involved in the ABA and ethylene signaling pathways. The phase transition from vegetative to reproductive growth is a complex biological process that is regulated by a wide range of genes that also interact with one another, leading to a cascade of physiological and biological changes. The functions of certain transcription factors, such as AP2, WRKY, MYB, MYC, NAC and CRT, during the regulation of flower bud differentiation should be further studied in chrysanthemum.

Genes involved in signal transduction as well as sugar, kinase, ubiquitin and defense pathways were also up-regulated in floral buds relative to vegetative buds and buds. These up-regulated genes were enriched for the GO categories ‘carbohydrate metabolic process,’ ‘protein kinase activity,’ ‘plant hormone signal transduction,’ and ‘defense response,’ among others. These findings indicate that genes involved in carbohydrate metabolic processes, protein kinase activities, plant hormone signal transduction, defense responses and other related processes play a role in floral differentiation in chrysanthemum. This hypothesis is also consistent with previous studies showing that 1) various phytohormones play an important role in the regulation of flowering in both long- and short-day plants; 2) the soluble sugar and starch contents of leaves and buds are temporarily increased in *Sinapis* during the induction of flowering; and 3) a calcium-dependent protein kinase is involved in photoperiodic flower induction in *Pharbitis nil*. [23, 24, 25].

In addition, there were many unigenes coding uncharacterized proteins that are also worthy of attention. Some of these were assigned to flower development-related GO terms, and many others were significantly up-regulated in floral buds, suggesting that they are involved in regulating chrysanthemum flower development. Further studies should be conducted to reveal the exact roles of these uncharacterized genes in the regulation of flower development.

The chrysanthemum floral meristem initiates in response to a single short day; the photoperiod is considered to be the most important factor controlling chrysanthemum flowering time. We identified many genes (*CRY*, *PHY*, *LHY*, *FKF1*, *TOC1*, *ZTL*, *GI*, *CO*, *FT*) encoding components of light signal transduction pathways or related to circadian rhythms. *LFY* expression leads to a series of transcriptional activities that control floral meristem formation and meristem identity [4]. The homologous gene (*LFYCL*) in chrysanthemum was identified. In addition, the homologs of AP2 and many MADS box genes involved in floral meristem identity were identified in chrysanthemum. According to the ABC model, A function genes (*AP1*, *AP2*) specify the identity of the sepal, A function genes together with B function genes (*AP3*, *PI*, and *TM6*) specify the petals, B function genes together with C function genes (*AG*) specify the stamen, and C function genes specify the carpel. However, the capitulum of chrysanthemum is composed of hundreds of individual florets which are specialized in structure and function, indicating that the regulatory mechanism of A, B and C function genes should be further studied in this species. The homologous genes of A, B and C function genes were identified in this study. These genes are important candidate genes for molecular cloning and functional analysis of flowering regulation genes in chrysanthemum.

## Conclusion

In this study, comparative transcriptome analysis revealed significant differences in gene expression and signaling pathways among the vegetative buds, floral buds and buds in *Chrysanthemum morifolium*. We found that genes involved in specific biological processes were activated and considerably up-regulated during the process of flower bud differentiation, including genes coding for important transcription factors, as well as genes involved in carbohydrate metabolic processes, protein kinase activities, plant hormone signal transduction and defense responses. These findings indicate that the phase transition from vegetative to reproductive growth is a complex biological process regulated by a wide range of factors, during which time many physiological processes, such as sugar metabolism and phytohormone regulation, become activated. The identification of homologous genes involved in the photoperiod pathway will likely contribute to studies of flowering-time regulation in chrysanthemum.

The transition from vegetative growth to the reproductive period in chrysanthemum is regulated by a large number of genes involved in complex molecular pathways. These results represent a first step towards illuminating the molecular mechanisms of flower development in chrysanthemum, and they will provide abundant genomic resources and new candidate genes for studies of flower-time regulation, breeding and molecular biology in this plant species.

## Materials and Methods

### Plant materials and RNA extraction

The tissues (vegetative buds, floral buds, buds) used in the study were obtained from a ground-cover chrysanthemum variety (*Chrysanthemum morifolium* ‘Fenditan’, a hybrid of chrysanthemum varieties) cultivated in a greenhouse under a 16-hr light/8-hr dark cycle for 180 days and then an 8-hr light/16-hr dark cycle at 23°C in Beijing Forestry University (116.3°E, 40.0°N). Under long-day treatment, 70 vegetative buds were collected between 9:00–12:00 a.m. When the short-day treatment began, 70 apical buds were collected between 9:00–12:00 a.m. every week until visible floral buds were formed. After floral buds were formed, 30–50 buds were collected between 9:00–12:00 a.m. every week until the ray florets started to show color. All plant tissues collected were placed immediately in liquid N_2_ and stored at −70°C until RNA was extracted. The individual plants used for collecting materials were all derived by tissue culture from the C. morifolium genotype ‘Fenditan.’ Total RNA was extracted using an RNAisomate RNA Easyspin Isolation System, and a NanoDrop ND2000 instrument was used to assess the RNA quantity and quality.

### Illumina sequencing, de novo assembly and functional annotation

To reveal differences in the chrysanthemum transcriptome before and after flower differentiation, we collected apical buds during the adult vegetative phase before short-day treatment. However, flower differentiation and bud development are continuous developmental process. Therefore, to obtain more complete transcriptome information, we pooled equivalent quantities of total RNA isolated from different developmental stages of flower differentiation and bud development, and the pooled total RNA samples were used for sequencing. In total, 10 μg total RNA was collected for each sample for sequencing. We conducted Illumina sequencing using an Illumina HiSeq 2000 system according to the manufacturer’s protocols with paired-end 2×100-nt multiplexing according to the manufacturer’s instructions.Briefly, poly (A) mRNA was purified using magnetic beads with oligo (dT) and was then fragmented into small pieces. After the first-strand cDNAs were synthesized using random hexamer primers, the second strand was synthesized using appropriate buffers, dNTPs, RNase H, and DNA polymerase I. After purification using the QiaQuick PCR extraction kit, the double-stranded cDNAs were resolved using EB buffer for end reparation and poly (A) addition. We then connected the short fragments with sequencing adapters. Finally, a cDNA library was constructed, which was sequenced using an IlluminaHiSeq 2000. Low-quality reads were removed using an in-house Perl script. The transcriptome sequences were then assembled with clean reads. First, the distinct contigs were assembled using the short reads with the software program CLC Genomic Workbench 5.5. Next, scaffolds between the contigs were constructed by employing the paired-end relationships between the reads. Finally, we filled the intra-scaffold gaps and constructed a non-redundant unigene set from the assembled datasets using CAP3 software [26].

For functional annotation, Blastx alignment (E-value\1.00E-5) was conducted between unigenes and protein databases, including Nr (non-redundant protein database, NCBI), Swiss-Port (http://www.expasy.ch/sprot), KEGG (http://www.genome.jp/kegg) and KOG (http://www.ncbi.nlm.nih.gov/KOG). The sequence directions of unigenes were identified according to the best alignment results. The Blast2GO program was used to produce GO (http://www.genome.jp/kegg) annotations for the assembled unigenes [27].

### Statistical analyses

The expression levels of the unigenes were calculated by RNA-Seq quantification analysis as the number of reads per kilobase of an exon region in a given gene per million mapped reads (RPKM) [28]. The transcriptomes of the three samples were used as a reference to screen and analyze the differentially expressed unigenes. A rigorous algorithm was used to identify DEGs using the method of Audic et al. (1997) [29]. The false discovery rate (FDR) was used to determine the threshold of the P-value in multiple tests and analyses [30]. A FDR of < 0.05 and an absolute value of log2 (ratio) ≥ 2 were the thresholds used to define significant differences in gene expression [28]. The DEGs with minimum two-fold changes in expression were subjected to differential gene expression analysis.

### Real-time quantitative PCR verification by qRT-PCR

Total RNA was extracted from the vegetative buds, floral buds and buds as described above. After treatment with DNase, the total RNA was subjected to reverse transcription to produce cDNA using a reverse transcription system. Real-time RT-PCR was conducted using the PikoReal real-time PCR system. Each reaction was performed in a total reaction mixture volume of 20 μL, which contained 2 μL of first-strand cDNA as template. The amplification program was: 30 s at 95°C, 40 cycles of 5 s at 95°C and 30 s at 60°C. Gene-specific primers (S12 Table) were designed to provide a relative quantification of each gene. All of the real-time RT-PCR experiments were conducted using three biological replicates, and all replicates were measured in triplicate. We analyzed the relative expression levels using the 2-ΔΔCt method, and the protein phosphatase 2A (PP2Acs) gene of *C*. *morifolium* was used as a reference gene [31].

## Supporting Information

S1 Table The unigenes assigned to the GO terms related to flower development. (XLSX)

S2 Table Up-regulated genes in floral buds relative to vegetative buds. (XLSX)

S3 Table Down-regulated genes in floral buds relative to vegetative buds. (XLSX)

S4 Table Genes down-regulated in buds relative to floral buds. (XLSX)

S5 Table Genes up-regulated in buds relative to floral buds. (XLSX)

S6 Table DEGs specifically expressed in buds relative to floral buds. (XLSX)

S7 Table The unigenes enriched in 'carbohydrate metabolic process' (GO0005975). (XLSX)

S8 Table The unigenes enriched in 'signal transduction’ (GO0007165). (XLSX)

S9 Table The unigenes enriched in ‘protein kinase activity’ (GO:0004672). (XLSX)

S10 Table The unigenes enriched in ' defense response' (GO:0006952). (XLSX)

S11 Table Homologous genes of the regulators involved in the photoperiod pathway in chrysanthemum. (XLSX)

S12 Table Primers used in qRT-PCR of *Chrysanthemum morifolium*. (DOCX)
