# Supplementary material for: Whole-Transcriptome Analysis of Differentially Expressed Genes in the Vegetative Buds, Floral Buds and Buds of Chrysanthemum morifolium
Source: PLoS One. 2015 May 26;10(5):e0128009. doi: 10.1371/journal.pone.0128009 (PMC4444331; doi:10.1371/journal.pone.0128009)
Supplement: S12 Table — (DOCX) [file pone.0128009.s012.docx]

**Table S12 Primers used in qRT-PCR of *Chrysanthemum morifolium* (RT-qPCR)**

| **Gene name** | | **Forward primer sequence (5′-3′)** | **Reverse primer sequence (5′-3′)** | **Correlation between RNA-Seq and qRT-PCR (R2)** |
| --- | --- | --- | --- | --- |
| MADS-boxCL1 | | TCTATCTTTCCCCTACCCAT | TTGCCAAGATTAAATGACCTG | 1 |
| MADS-boxCL2 | | AACTTATGTATTTGGTGCAAC | TAAGCAAACCGTTTACATCC | 0.99 |
| MADS-boxCL3 | | TCTTCAATACTCATGCCAC | ATTATTCCTTATCGGTAGAGC | 0.99 |
| GDEF1CL | | ATGCAAGATTCACTAACCGTA | AGCCATAGAGATTTGATACACC | 0.99 |
| AP2/ERFCL3 | | ATATGATAAAACCACAGCCAGT | CACCCCACTTTCTAGGTCA | 0.99 |
| CRTCL | | GTGCCGACTTTTAATCAGACA | GAAGGCATACTACCCAGCAAG | 1 |
| MYBCL1 | | TTGACATTTTATTGGCGGTA | TCATTTAAGCCCCAAGGTG | 0.99 |
| DoubleWRKYCL | | ACTCAAGGGATACAAATGGCTA | ACCCGATTTGAGAATGCACA | 0.99 |
| GA2O2CL | | CTCTAGAATCTCACATGCCAT | TTCTCAACCTTAAGCCCGAA | 1 |
| ETHR3CL | | TAATCCACTCCAGACGACGAA | ACCCGATCTATCAAATGGAAC | 0.99 |
| UnknownC1 | | CCTCCACTAGGTTTCTTGTTA | ACCCTCATGGAAGTGTGAAAG | 0.97 |
| UnknownC2 | | CTGATTGTAACACCGAAACTG | GGCTTGACATCATGAGCTGTG | 0.98 |
| UnknownC3 | | CACAAATTGCTAATCCACTTG | CTTGGTCACATCTAACATAGC | 0.98 |
| UnknownC4 | | CTCAAACTTGAACGTTGTTGG | CAAGTTTGAGAGACTTCAGCT | 0.99 |
| UnknownC5 | | CCTTGACATCGTTCAGTTAGC | GTGACACCTATCCTCTTTGGG | 0.97 |
| UnknownC6 | | CATGGTGGTTTGTGGAAGTGC | AGCCAAACGAAACCTAAAATG | 0.97 |
| UnknownC7 | | GCCTCTTCACCAGCTATCTTC | CCACTCAGCTTAAACACTTGA | 0.99 |
| UnknownC8 | | GTTTTGCTGATGAGTACACAC | CCAAAAATCTTAACTGGCTCG | 0.99 |
| UnknownC9 | | CCTCATGTTGTATTTTTCGCC | TCTATATTGCTAAAGCCACCG | 0.97 |
| UnknownC10 | | CTAAAAGCTTGATCAGAGCCA | AGCAACAACACACTCATCACC | 0.98 |
| PP2Acs（Reference gene） | | ATCAGAACAGGAGGTCAGGG | TAATTTGTATCGGGGCACTT |  |
|  |  |  |  |  |
|  |  |  |  |  |
|  |  |  |  |  |
|  |  |  |  |  |
|  |  |  |  |  |
|  |  |  |  |  |
|  |  |  |  |  |
|  |  |  |  |  |
|  |  |  |  |  |
|  |  |  |  |  |
|  |  |  |  |  |
|  |  |  |  |  |
|  |  |  |  |  |
|  |  |  |  |  |
|  |  |  |  |  |
|  |  |  |  |  |
|  |  |  |  |  |
|  |  |  |  |  |
|  |  |  |  |  |
